# Effect of catch-up sleep on obesity in Korean adolescents: a nationwide cross-sectional study

**DOI:** 10.3389/fped.2023.1213558

**Published:** 2023-07-26

**Authors:** Youngha Choi, Sujin Kim, Myeongseob Lee, Hae In Lee, Kyungchul Song, Junghwan Suh, Hyun Wook Chae, Ho-Seong Kim, Ahreum Kwon

**Affiliations:** ^1^ Department of Pediatrics, Kangwon National University Hospital, Chuncheon, Republic of Korea; ^2^ Department of Pediatrics, Severance Children’s Hospital, Institute of Endocrinology, Yonsei University College of Medicine, Seoul, Republic of Korea; ^3^ Department of Pediatrics, CHA Gangnam Medical Center, CHA University, Seoul, Republic of Korea

**Keywords:** sleep deprivation, sleep duration, overweight, obesity, adolescents, catch-up sleep

## Abstract

**Background:**

Adolescents have weekday/weekend sleep discrepancies and may compensate for weekday sleep debt through sleep extension on weekends.

**Objective:**

We investigated the effects of total sleep duration on weekdays/weekends on obesity and determined if weekend catch-up sleep has an ameliorating effect on obesity in Korean adolescents.

**Methods:**

Using data from the KNHANES VII, 1,306 middle and high school students were assessed for total sleep duration on weekdays, weekends, and the entire week, as well as weekend sleep extension. Participants were classified into four groups according to weekend sleep extension.

**Results:**

Total sleep duration and weekend sleep duration were negatively associated with body mass index z-score. Increased weekend sleep duration and sleep extension on weekends decreased the relative risk of overweight/obesity with each 30 min increment, reducing the risk by a factor of 0.39 and 0.93, respectively. The risk of overweight/obesity in adolescents who slept less than 6 h on weekdays increased by a factor of 1.93 when they slept for less than 3 h on weekends.

**Conclusion:**

Weekend catch-up sleep had a negative dose-dependent association with obesity in Korean adolescents. Sleeping longer on weekends may be associated with a decreased risk of obesity, even if the adolescent obtains less sleep during weekdays. However, further prospective studies are needed to establish the causality between extended weekend sleep and obesity.

## 1. Introduction

The World Health Organization (WHO) has recently reported that between 1975 and 2016, the prevalence of overweight/obesity among children and adolescents has increased more than four times (1). While there are a number of risk factors associated with adolescent overweight/obesity, it is well established that sleep problems, and especially short sleep duration, are correlated with the development of overweight/obesity (2–8). Importantly, over the last century, 20 countries have reported a decline in sleep duration in adolescents (9, 10), and as a result, millions of adolescents worldwide currently achieve less than 8 h sleep per night, especially on weekdays (11, 12).

Adolescents suffer sleep deprivation due to psychosocial factors including academic demands, social activity, screen time, and social media. Weekday sleep duration is particularly shorter than that on weekends due to school hours, which creates a “sleep debt” (9–11, 13–17). This sleep debt can be compensated for over the weekend through “catch-up sleep” (13, 17–20). Previous studies have shown that weekend catch-up sleep is associated with decreased risk of being overweight/obesity or metabolic syndrome (21–25), but some studies reported that it does not ameliorate the risk of obesity or was not associated with body weight (26–29).

Therefore, we aimed to evaluate whether catch-up sleep over the weekend can decrease the risk of overweight/obesity among Korean middle school and high school students. Additionally, we aimed to determine the required number of hour necessary to mitigate the risk of obesity caused by inadequate sleep on weekdays, taking into consideration the duration of sleep during weekdays.

## 2. Materials and methods

### 2.1. Data sources and study population

Data from the 7th Korea National Health and Nutrition Examination Survey (KNHANES VII), conducted between 2016 and 2018, were analyzed. KNHANES has been conducted periodically since 1998 by the Korea Centers for Disease Control and Prevention and is a large cross-sectional representative survey of the health and nutritional status of the South Korean population. Informed consent was obtained from all participants for the survey, and for this study, data from adolescents who were middle school students and high school students were included. The institutional review board of Kangwon National University Hospital approved the study protocol (approval number KNUH-2022-10-001).

**Figure 1:**
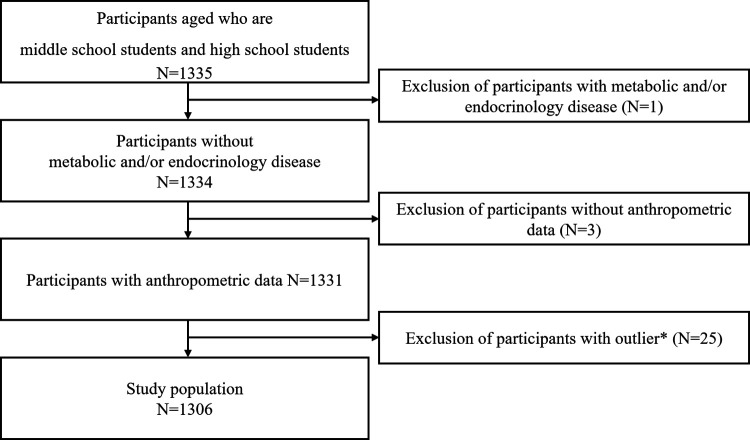
Selection of study participants. Participants with a calculated sleep duration on weekdays of less than 4 h (*N* = 9) or more than 14 h (*N* = 1), a calculated sleep duration on weekends of less than 4 h (*N* = 3) or more than 15 h (*N* = 1), a bedtime on weekdays after 4 a.m. (*N* = 0), or a wake-up time on weekdays after 10 a.m. (*N* = 11) are treated as outliers (total *N* = 25), and excluded.

Of the 1,335 middle school students and high school students from KNHANES VII, those who had metabolic and endocrine diseases diagnosed by a doctor (*N* = 1) and those without records of anthropometric data (*N* = 3) were excluded. Participants were considered to be outliers if they: (i) had a calculated sleep duration on weekdays exceeding 14 h (*N* = 1) or <4 h (*N* = 9); (ii) had a calculated sleep duration on weekend exceeding 15 h (*N* = 1) or <4 h (*N* = 3); (iii) went to bed after 4 a.m. (*N* = 0); or (iv) woke up after 10 a.m. (*N* = 11) on weekdays, and were subsequently excluded. As such, the final number of study participants was 1,306 (Figure 1).

### 2.2. Anthropometry

The KNHANES VII survey included anthropometric data for each participant, including age, sex, height, weight, and body mass index (BMI). A portable stadiometer was used to measure height to the nearest 0.1 cm, and a digital scale was used to measure weight to the nearest 0.1 kg, with the participants wearing light clothing and no shoes. BMI was calculated as weight (in kilograms) divided by the square of height (in meters), and standard scores (z-scores) for BMI were obtained for the same age and sex from the 2017 Korean Children and Adolescents Growth Chart. Underweight, overweight, and obesity were defined as a BMI at the 5th percentile or below, between the 85th and 95th percentiles, and at the 95th percentile or above, respectively, for the same age and sex. The four categories of weight status were then combined to create dichotomous variables (underweight/normal weight and overweight/obesity).

### 2.3. Sleep assessment

Adolescents were asked to report their sleep habits while at home on weekdays and weekends. The questionnaires used to assess sleep were developed for individuals aged 12 years and older by the Korea Centers for Disease Control and Prevention (Supplementary Figure S1). The variables of interest were: (i) sleep duration, (ii) bedtime, (iii) wake time, and (iv) sleep mid-time. Bedtime was defined as the time that the participants fell asleep at the end of the day, and wake time was defined as the time when they awoke from this sleep (i.e., daytime naps were not included). The values for bedtime and wake time were converted into minutes to calculate sleep duration and sleep mid-time, in addition to analyzing their effect on obesity as continuous variables. Bedtime and wake time were calculated from midnight; for example, when a participant went to bed at 10 pm and woke up at 7 a.m., it would be stated as bedtime at 22 × 60 = 1,320 min and waking up at 7 × 60 = 420 min. However, if the bedtime was after midnight, it was calculated by adding 24 h; that is, a person who began to sleep at 1 a.m. would have bedtime stated as (24 + 1) × 60 = 1,500 min. Total sleep duration (TSD) was calculated by considering bedtime and wake time. The TSD for the entire week was a weighted mean of sleep durations on weekdays and weekends, calculated as:

(5×TSDonweekdays+2×TSDonweekends)/7

Participants were classified into four groups as follows: (i) less than 6 h, (ii) 6–7 h, (iii) 7–8 h, and (iv) 8 h or more according to TSD per weekday.

Weekend sleep extension (WK-E) was defined as the difference in sleep duration between weekdays and weekends and was calculated as TSD on weekend minus TSD on weekday. Participants were then classified into four groups based on WK-E as follows: (i) less than 1 h, (ii) 1–2 h, (iii) 2–3 h, and (iv) 3 h or more. To evaluate the effect of weekday and weekend sleep on TSD, the participants were classified into one of four sleep duration groups, using the median values for TSD on weekdays and weekends: (i) short sleep on both weekdays and weekends (SWD/SWK), (ii) long sleep on weekdays and short sleep on weekends (LWD/SWK), (iii) short sleep on weekdays and long sleep on weekends (SWD/LWK), and (iv) long sleep on both weekdays and weekends (LWD/LWK).

### 2.4. Covariates

The covariate data collected from the lifestyle questionnaire included sex, physical activity, sedentary time, food intake, and household income. Physical activity was determined by the number of days the participant exercised at an intensity, which resulted in heavy breathing for >60 min in a week, both during and outside of school hours. The participants also reported sedentary time as the time (h and min) spent sitting or lying down each day during the past week. Food intake was assessed by categorizing foods into grains, bean products, potatoes, meat, fish, seaweed, fruit, vegetables, milk products, and beverages. Participants were required to recall the amount of each food type eaten during the previous 2 days. Nutritionists assessed this food intake and converted it into weight in grams and calories consumed. The cumulative income of the family was partially adjusted based on the number of family members and was divided into quintiles according to the average monthly household income.

### 2.5. Statistical analyses

Statistical analyses were performed using SPSS (Version 26.0; IBM Corp., Armonk, NY, USA) and SAS version 9.4 (SAS Inc., Cary, NC, USA). All tests were two-tailed, with a significance level set at 0.05. SPSS and SAS survey procedures were used to describe the complex sampling design in KNHANES. Sample weights were assigned to participants to represent the adolescent population of South Korea from 2016 to 2018 for all analyses. Sample weights were generated by accounting for the complex sample design, which consisted of non-response rates of the target population, multistage stratification, and posterior stratification. The normality of data distribution of variables was examined using univariate and histogram methods, and frequency tests were used to assess the relationships between variables.

The mean values of sleep dimensions and other variables were compared between the underweight/normal weight group and the overweight/obesity group using complex samples general linear model (CSGLM) for continuous variables and Rao–Scott *χ*^2^ test for categorical data. The potential association between each continuous sleep variable and BMI z-score was investigated using CSGLM. Additionally, the relative risk of overweight/obesity conferred by each sleep variable was assessed using Complex Samples Poisson Regression Analysis. The mean BMI z-score according to TSD on weekdays and WK-E were also investigated using CSGLM. The mean BMI z-score and the risk of overweight/obesity among the four sleep duration groups were compared using CSGLM and Complex Samples Poisson Regression Analysis, respectively. Trends were tested to assess the decremental mean BMI z-score across the four groups, and the relative risk for the overweight/obesity group was calculated using the SWD/LWK group as the reference. Various models were developed for CSGLM and Complex Samples Poisson Regression Analysis. Model 1 was developed without adjustments for covariates. Model 2 was adjusted for physical activity, sedentary time, household income, total food intake (kJ), and frequency of breakfast intake. All data were reported as mean ± standard error (SE) or *n* (%).

## 3. Results

Comparison of baseline characteristics between participants in the underweight/normal weight and overweight/obesity groups.

A total of 1,306 participants (mean age, 15.4 ± 0.1 years; males, 52.5%) were included in the study (Figure 1) with their characteristics presented in Table 1. The mean TSD on weekdays and weekends were 6.8 h (SE, 2.4 min) and 8.7 h (SE, 3.3 min), respectively, and the mean TSD of the whole week was 7.4 h (SE, 2.2 min). WK-E was determined to be 1.8 h (SE, 3.4 min). Importantly, significant differences between groups with underweight/normal weight and overweight/obesity were observed for TSD on weekends, TSD whole week, wake time on weekends, and WK-E. Overall, the participants in the underweight/normal weight group woke up later and slept more on weekends than those in the overweight/obesity group.

**Table 1:** Characteristics of study participants included in the analysis stratified by weight status*.

	Total analysis group	Underweight/normal weight	Overweight/obese	*p*-value
	*N* = 1,306	*N* = 105/911^†^	*N* = 124/166^†^	
	Mean (SE)	Mean (SE)	Means (SE)	
Age	15.4 (0.1)	15.3 (0.1)	15.5 (0.1)	0.187
Sex, male, *n* (weighed)	687 (52.5%)	517 (51.9%)	161 (54.3%)	0.183
Height, cm	166.1 (0.3)	**165.8** **(0.3)**	**167.3** (**0.6)**	**0**.**020**
Weight, kg	59.6 (0.4)	**55.0** (**0.3)**	**76.3** (**0.9)**	**<0**.**001**
Waist circumference	71.9 (0.3)	**68.2** (**0.2)**	**85.4** (**0.7)**	**<0**.**001**
BMI	21.5 (0.1)	**20.0** (**0.1)**	**27.1** (**0.2)**	**<0**.**001**
BMI z-score	0.13 (0.04)	**−0.42** (**0.03)**	**2.15** (**0.07)**	**<0**.**001**
Household income, *n* (weighted percent)				0.192
Q1	116 (9.2)	84 (8.5)	32 (11.8)	
Q2	211 (16.5)	158 (16.4)	53 (16.9)	
Q3	302 (22.8)	241 (23.5)	61 (20.2)	
Q4	351 (27.5)	269 (26.7)	82 (30.6)	
Q5	324 (24.0)	263 (24.9)	61 (20.5)	
Sedentary time, h	11.3 (0.1)	11.3 (0.1)	11.4 (0.2)	0.676
Anerobic exercise, (days/week), *n*				0.185
Not at all	831 (62.0)	642 (61.7)	189 (63.3)	
1 day	146 (11.6)	111 (11.2)	35 (13.2)	
2 days	109 (8.6)	93 (9.3)	16 (6.4)	
3 days	87 (6.9)	64 (6.8)	23 (7.4)	
4 days	37 (2.6)	31 (2.7)	6 (2.2)	
More than 5 days	96 (8.2)	75 (8.5)	21 (7.5)	
Sleep duration, h				
TSD WD	6.8 (0.0)	6.9 (0.0)	6.8 (0.1)	0.313
TSD WK	8.7 (0.1)	**8.8** (**0.1)**	**8.4** (**0.1)**	**0**.**001**
TSD whole week	7.4 (0.0)	**7.4** (**0.0)**	**7.2** (**0.1)**	**0**.**039**
Sleep time, h:min				
BT WD	00:13 (2.6)	00:13 (2.6)	00:14 (5.1)	0.903
BT WK	00:51 (2.8)	00:49 (2.9)	00:57 (6.9)	0.279
WT WD	07:04 (1.9)	07:05 (2.0)	07:00 (2.9)	0.116
WT WK	09:36 (4.1)	**09:39** (**4.6)**	**09:23** (**7.2)**	**0**.**038**
Sleep differences, h				
WK-E	1.8 (0.1)	**1.9** (**0.1)**	**1.6** (**0.1)**	**0**.**012**

SE, standard error; BMI, body mass index; TSD, total sleep duration; WD, weekdays; WK, weekends; BT, bedtime; WT, wake time; WK-E, weekend sleep extension.

* All data were weighted values considering the complex sample design.

^†^ Categorization according to BMI z-score. Data are presented as mean (SE) or *n* (weighted %).

Significant results are shown in bold. TSD whole week is calculated as (WD × 5 + WK × 2)/7.

### 3.1. Association between sleep variables and body weight

Results of the linear regression models are summarized in Table 2. The sequential regression models for each sleep variable showed a significant negative correlation with the BMI z-score, as did the mean TSD for weekdays, weekends and whole week, particularly on weekends, even after adjusting for specific variables. Due to the positive correlations between sleep duration and wake-up time (weekdays, *β* = 0.707, *p* < 0.001; weekends, *β* = 0.061, *p* < 0.001), there was also a negative correlation between wake-up time and BMI z-score on both weekdays and weekends (Table 2). Moreover, the longer WK-E was negatively correlated with BMI z-score (Table 2).

**Table 2:** Linear regression analyses to assess the potential association between variables and BMI, and poisson regression analyses to assess the relative risk between variables and overweight/obesity*^†^.

	Model 1					Model 2				
	β	*p*-value	RR	95% CI		β	*p*-value	RR	95% CI	
Sleep duration, h										
TSD WD	**−0** **.** **04**	**0**.**025**	0.97	0.92	1.03	**−0**.**05**	**0**.**029**	0.97	0.89	1.05
TSD WK	**−0**.**50**	**<0**.**001**	**0**.**47**	**0**.**30**	**0**.**75**	**−0**.**56**	**<0**.**001**	**0**.**39**	**0**.**22**	**0**.**70**
TSD whole week	**−0**.**06**	**0**.**001**	**0**.**93**	**0**.**88**	**1**.**00**	**−0**.**09**	**0**.**001**	**0**.**90**	**0**.**82**	**0**.**99**
Sleep time, h:min										
BT WD	0.02	0.243	1.00	0.95	1.06	0.02	0.454	0.98	0.90	1.07
BT WK	0.02	0.102	1.03	0.97	1.10	0.02	0.349	1.01	0.94	1.09
WT WD	**−0**.**07**	**0**.**029**	0.91	0.80	1.02	**−0**.**10**	**0**.**009**	**0**.**85**	**0**.**74**	**0**.**98**
WT WK	**−0**.**03**	**0**.**016**	**0**.**96**	**0**.**92**	**1**.**00**	**−0**.**04**	**0**.**004**	**0**.**93**	**0**.**88**	**0**.**98**
Sleep discrepancy, h										
WK-E	**−0**.**03**	**0**.**011**	**0**.**95**	**0**.**91**	**0**.**98**	**−0**.**03**	**0**.**011**	**0**.**93**	**0**.**89**	**0**.**98**

RR, relative risk; CI, confidence interval; TSD, total sleep duration; WD, weekdays; WK, weekends; BT, bed time; WT, wake time; WK-E, weekend sleep extension. Model 1: unadjusted. Model 2: adjusted for physical activity, sedentary time, household income, and total intake (kcal).

* All data are weighted values based on the complex sample design. 1 unit, 30 min.

^†^ Relative risk indicates the risk of overweight/obesity.

Significant results are shown in bold.

Table 2 also presents the Poisson regression analysis between sleep variables and the relative risk of overweight/obesity. Importantly, longer TSD on weekends and longer WK-E decreased the relative risk of overweight/obesity. Each 30 min increase in sleep over the weekend reduced the relative risk of overweight/obesity by a factor of 0.39, regardless of covariates. In addition, as the WK-E increased by 30 min, the risk of overweight/obesity decreased by a factor of 0.93, following adjustment of the covariates. These results suggest that longer TSD on weekends and getting more sleep on weekends than weekdays may be associated with a reduction in the mean BMI z-score and relative risk of overweight/obesity.

### 3.2. Association of body weight with the four sleep duration groups on weekdays and weekends in adolescents

**Figure 2:**
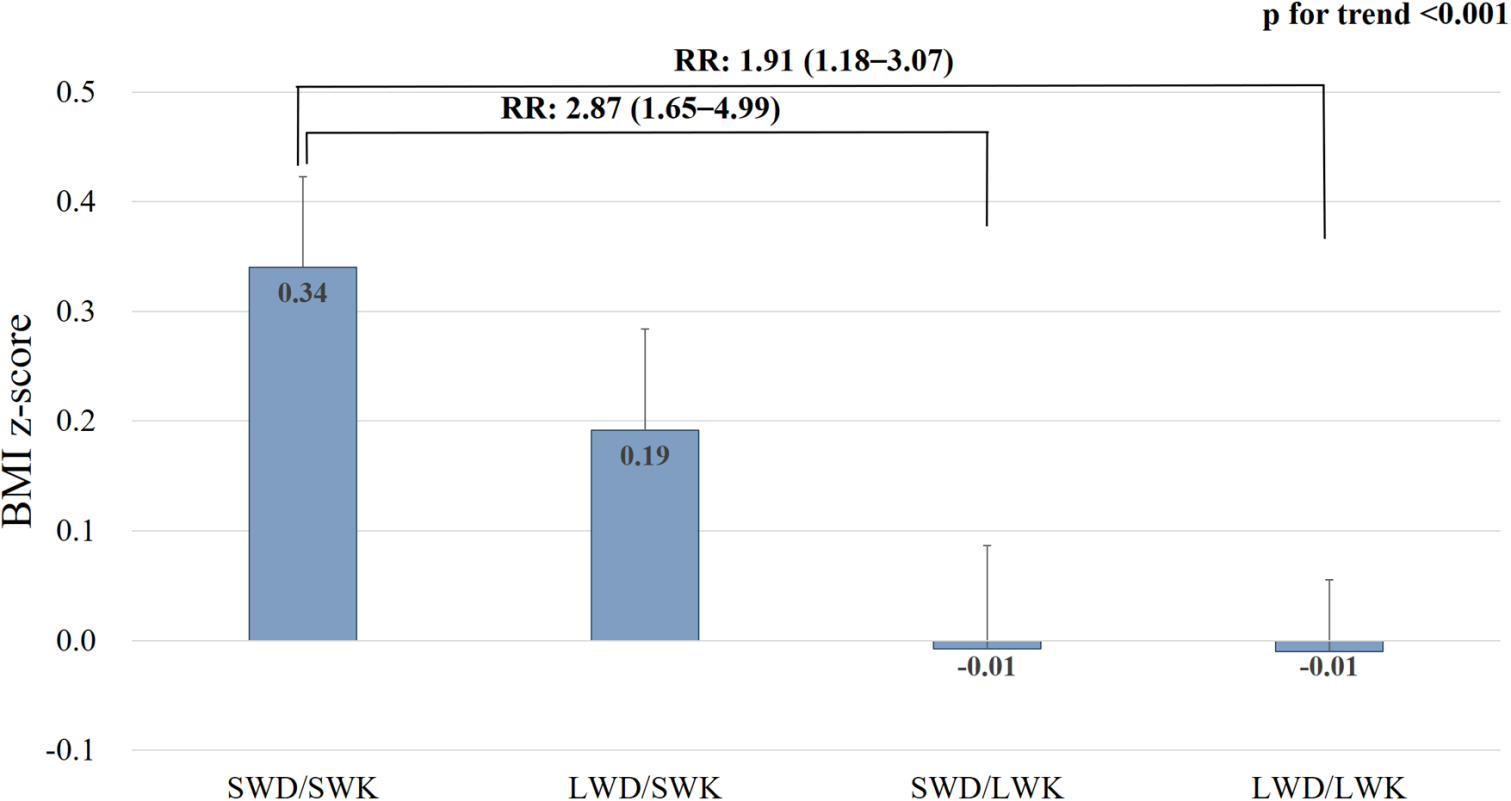
Body mass index z-score and relative risk of overweight/obesity in the four sleep duration groups. SWD, short sleep on weekday; SWK, short sleep on weekends; LWD, long sleep on weekdays; LWK, long sleep on weekends; BMI, body mass index; RR, relative risk.

To evaluate whether there was a difference in the effects of TSD between weekdays and weekends on overweight/obesity, the participants were divided into four sleep duration groups (SWD/SWK, LWD/SWK, SWD/LWK, and LWD/LWK) and compared. Supplementary Table S1 shows the characteristics of the four groups, while Figure 2 shows the mean BMI z-score and relative risk of overweight/obesity. Those who slept less on weekends had a higher BMI z-score than those who slept longer on weekends, and the mean BMI z-score was the highest in SWD/SWK, while the lowest in LWD/LWK (Figure 2). Moreover, despite LWD/SWK sleeping more during the whole week than SWD/LWK (7.6 h and 7.0 h, respectively, Supplementary Table S1), the mean BMI z-score was significantly higher in LWD/SWK than that in SWD/LWK (Figure 2). After adjusting the variables, the participants in the SWD/SWK group were 2.87 and 1.91 times more likely to show overweight/obesity than those in the SWD/LWK group and LWD/LWK, respectively (Figure 2). These results demonstrated that longer sleep on weekends is important to reduce the mean BMI z-score and the relative risk of overweight/obesity.

### 3.3. Number of hours necessary to mitigate the relative risk of overweight/obesity taking into consideration the total sleep duration on weekdays

**Figure 3:**
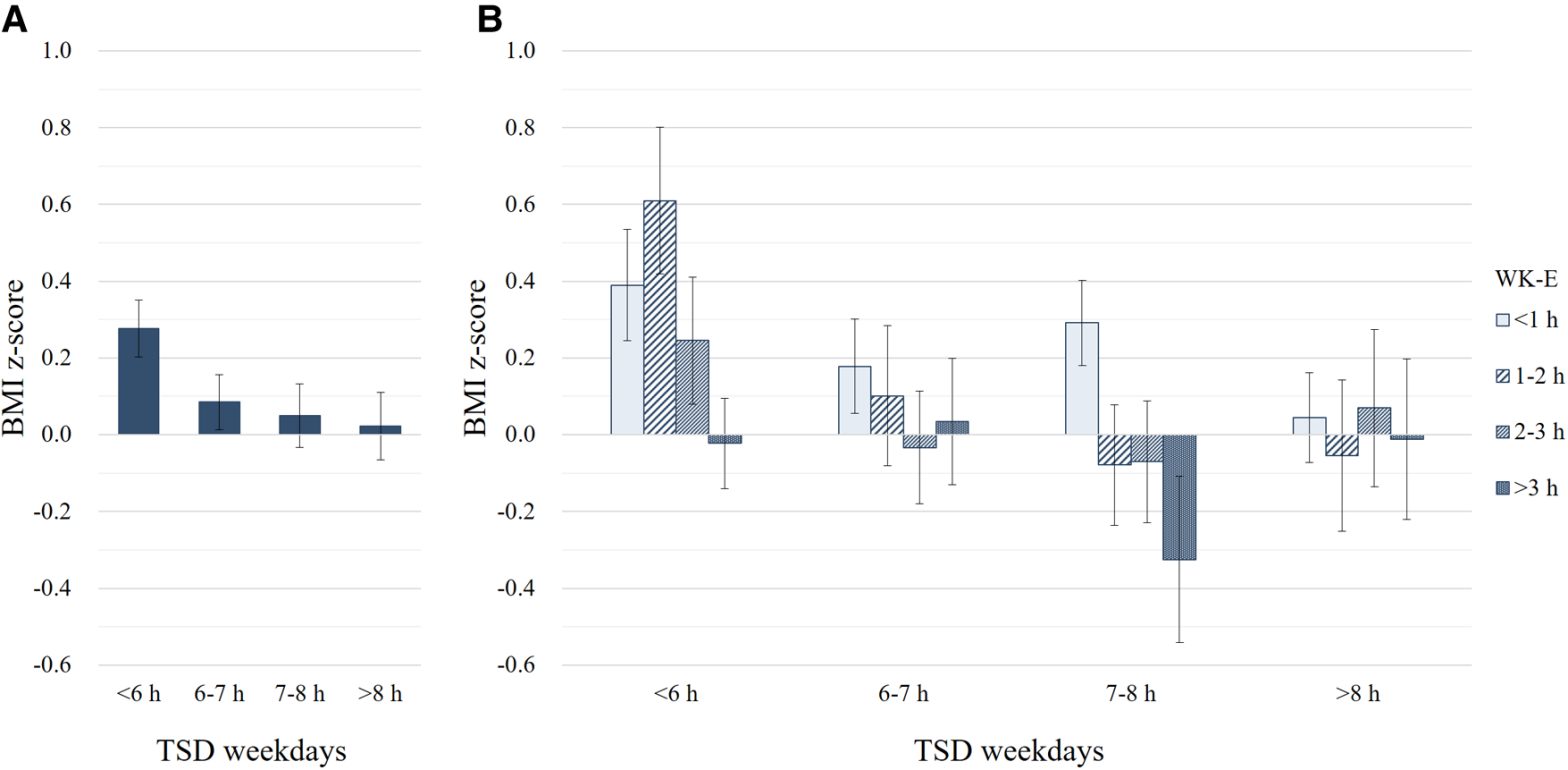
Effect of weekend sleep extension (WK-E) according to total sleep duration (TSD) on weekdays on body mass index (BMI) z-score. (**A**) BMI z-scores according to TSD on weekdays. (**B**) BMI z-scores according to TSD on weekdays and WK-E.

We evaluated how many sleep hour were required on weekends to reduce the relative risk of overweight/obesity. Participants with WK-E less than 2 h had an overweight/obesity risk 1.53 times [95% confidence interval (CI): 1.12–2.10] higher than participants with WK-E of 2 h or more (*p* = 0.008). To evaluate how much WK-E was required, the TSD on weekdays was classified into four groups: (i) less than 6 h, (ii) 6–7 h, (iii) 7–8 h, and (iv) more than 8 h. Similarly, WK-E was classified into four groups: (i) less than 1 h, (ii) 1–2 h, (iii) 2–3 h, and (iv) more than 3 h. The results are shown in Figure 3. The shorter the TSD on weekdays, the higher was the mean BMI z-score (Figure 3A). In participants with a TSD of less than 6 h on weekdays, BMI z-scores were less than 0 only when WK-E was more than 3 h (Figure 3B). Furthermore, when the WK-E was less than 3 h, the overweight/obesity relative risk increased by 1.93 times (95% CI, 1.07–3.48) in participants whose TSD on weekdays was less than 6 h. The BMI z-score was 0 or less when WK-E was more than 2 h for 6–7 h of TSD on weekdays and 1 h for 7–8 h of TSD on weekdays. Based on this, the shorter duration of sleep during weekdays was associated with a need for longer duration of sleep on weekends to achieve a BMI z-score of 0 or less. Interestingly, participants who slept more than 8 h on weekdays had near-zero BMI z-scores, regardless of their weekend sleep extension (Figure 3B). These results suggest that if the sleep duration is less than 8 h on weekdays, an additional hour of weekend sleep may be necessary for each hour of weekday sleep lost to reduce the BMI z-score.

## 4. Discussion

In the present study, we demonstrated that catch-up sleep on the weekend may be associated with a reduction in the mean BMI z-score and the relative risk of overweight/obesity by compensating for the short sleep duration during the weekdays in Korean adolescents. Even as little as an extra 30 min of sleep on weekends may decrease the risk of overweight/obesity by 61%. Moreover, as the sleep duration during weekdays decreases, an additional hour of weekend sleep may be necessary to reduce the BMI and the relative risk of overweight/obesity.

It is well known that short sleep duration increases the risk of obesity in adolescents (2, 3, 30–33). This study also found a significant correlation between TSD throughout the week and BMI z-score, as well as the relative risk of overweight/obesity in adolescents. Interestingly, weekend sleep duration showed a stronger correlation with BMI and the risk of overweight/obesity than weekday sleep duration. Furthermore, adolescents who sleep less on weekdays could prevent weight gain by sleeping more on weekends. In the present study, when the TSD on weekdays is fall below 8 h, an extra hour of sleep on weekends may be required to decrease the relative risk of overweight/obesity. For example, adolescents who sleep 7–8 h on weekdays need an additional hour of sleep on weekends, while adolescents who sleep 6–7 h on weekdays need an additional 2 h of sleep on weekends. In particular, adolescents who sleep less than 6 h on weekdays need an extended sleep of 3 h or more on weekends to reduce the risk of overweight/obesity. If such additional sleep is not obtained, the relative risk of obesity increases by 1.93 times.

Adolescents worldwide are not getting enough sleep (9–12). To compensate for the sleep debt incurred during weekdays, adolescents replenish their sleep on weekends (21–24, 34, 35). Korean adolescents in the present study do not get enough sleep on weekdays (6.8 h) and compensate for their sleep deficit by sleeping 1.4 h more on weekends. Such weekend additional sleep was found to be helpful in reducing the risk of overweight/obesity in adolescents in this study. Even though it remains controversial, previous studies also reported that catch-up sleep on weekends decreases BMI among adults who sleep insufficiently on weekdays (21–24). Furthermore, more catch-up sleep on weekends corroborates with lower BMI and lower risk of being overweight, and this occurs in a dose-dependent manner. Further, catch-up sleep on weekends tended to modify the obesity risk due to sleep insufficiency on weekdays (21–24).

There are several hypotheses as to why weekend catch-up sleep affects reducing the risk of overweight/obesity. Firstly, weekend catch-up sleep might help to replenish sleep debt and counteract the negative effects of sleep deprivation experienced by individuals with insufficient sleep on weekdays (22). Mechanically, catch-up sleep has been shown to have a positive effect on overweight/obesity by functionally improving the levels of several metabolic and endocrine variables and by counteracting the negative effects of sleep deprivation during the week. Short sleep can decrease leptin secretion through sympathetic activation (36), which can lead to a decrease in satiety. Catch-up sleep on weekends can prevent an increase in sympathetic tone, decrease β-cell responsiveness, and reduce non-insulin-dependent glucose uptake (23, 37). Additionally, catch-up sleep on weekends compensates for the metabolic disadvantages caused by sleep deprivation during weekdays (22). Specifically, catch-up sleep aids in recovery from the effects of oxidative stress and the inflammatory response, particularly the highly sensitive C-reactive protein level (38, 39).

This study is significant as it analyzed the relationship between weekend catch-up sleep and adolescent obesity using KNHANES data. Importantly, as these data were obtained from a large survey sample and weighted to represent the general population, these findings are applicable across the general population of Korean adolescents. Despite these strengths, our study still had some limitations. The results of this study are based on data obtained from self-reported questionnaires. Therefore, it is possible that the sleep duration and amount of weekend catch-up sleep reported may have been under- or over-estimated. Despite this, a prior study indicated that there were moderate correlations between actigraphy measurements, sleep diaries, and self-reported questionnaires in adolescents, suggesting that adolescents have the ability to accurately recall their typical sleep patterns (40). Considering the costs, polysomnography and actigraphy could not be used in our study, similar to that in other large population-based studies. Second, the study's cross-sectional design did not allow us to establish a clear causal relationship between weekend catch-up sleep and overweight/obesity. Therefore, we suggest that future prospective studies are required to determine the causality effectively.

In conclusion, catch-up sleep on weekend should be considered as an independent factor in epidemiological studies examining overweight/obesity. This study provides evidence that weekend catch-up sleep is associated with a dose-dependent decrease in the risk of overweight/obesity among Korean adolescents. The optimal sleep duration on weekdays to prevent overweight/obesity may be 8 h. Additionally, longer weekend catch-up sleep has been shown to be associated with a lower risk of obesity in adolescents, particularly when sleep duration during the weekdays is less than 8 h. Therefore, to aid in the prevention of overweight/obesity in adolescents with weekday sleep debt, it is important to obtain sufficient weekend catch-up sleep.

## Data Availability

The datasets presented in this study can be found in online repositories. The names of the repository/repositories and accession number(s) can be found below: KNHANES data are publicly available and can be accessed online (http://knhanes.kdca.go.kr/knhanes/eng/index.do).
