# Supplementary material for: Effect of catch-up sleep on obesity in Korean adolescents: a nationwide cross-sectional study
Source: Front Pediatr. 2023 Jul 26;11:1213558. doi: 10.3389/fped.2023.1213558 (PMC10410257; doi:10.3389/fped.2023.1213558)
Supplement: Supplementary file 1 [file Datasheet1.pdf]

Supplementary Material

Effect of Catch-up Sleep on Obesity in Korean Adolescents: A Nationwide Cross-sectional Study

Youngha Choi, Sujin Kim, Myeongseob Lee, Kyungchul Song, Junghwan Suh, Hyun Wook Chae, Ho-Seong Kim, Ahreum Kwon*

*** Correspondence: Ahruem Kwon**: armea@yuhs.ac

1 Supplementary Figures and Tables

1.1 Supplementary Figure S1. The Sleep Questionnaire (Original Korean Version)

You can access this questionnaire at Korea National Health & Nutrition Examination Survey "https://knhanes.kdca.go.kr"

2-13. 정신건강

문항번호	변수유형	변수명	변수설명	내용
1	N(2)	BP16_11	(만12세이상)<br>주중 잠자리에 든 시각_시	□□시<br>88. 비해당(소아)<br>99. 모름, 무응답
1	N(2)	BP16_12	(만12세이상)<br>주중 잠자리에 든 시각_분	□□분<br>88. 비해당(소아)<br>99. 모름, 무응답
1	N(2)	BP16_13	(만12세이상)<br>주중 일어난 시각_시	□□시<br>88. 비해당(소아)<br>99. 모름, 무응답
1	N(2)	BP16_14	(만12세이상)<br>주중 일어난 시각_분	□□분<br>88. 비해당(소아)<br>99. 모름, 무응답
2	N(2)	BP16_21	(만12세이상)<br>주말 잠자리에 든 시각_시	□□시<br>88. 비해당(소아)<br>99. 모름, 무응답
2	N(2)	BP16_22	(만12세이상)<br>주말 잠자리에 든 시각_분	□□분<br>88. 비해당(소아)<br>99. 모름, 무응답
2	N(2)	BP16_23	(만12세이상)<br>주말 일어난 시각_시	□□시<br>88. 비해당(소아)<br>99. 모름, 무응답
2	N(2)	BP16_24	(만12세이상)<br>주말 일어난 시각_분	□□분<br>88. 비해당(소아)<br>99. 모름, 무응답
	N(4)*	Total_slp_wk	(만12세이상)<br>주중 하루 평균 수면시간	□□□□분<br>8888. 비해당(소아)<br>9999. 모름, 무응답
	N(4)*	Total_slp_wd	(만12세이상)<br>주말 하루 평균 수면시간	□□□□분<br>8888. 비해당(소아)<br>9999. 모름, 무응답

* 생성변수
** 정신건강 자료는 HNYR_ALL (YR은 해당연도 두자리수) DB에 포함

설문지

1. 평소, 주중(또는 일하는 날)에 잠자리에 든 시각과 일어난 시각은 언제입니까?

① 잠자리에 든 시각 □□시 **(BP6_11)** □□분 **(BP6_12)**
② 일어난 시각 □□시 **(BP6_13)** □□분 **(BP6_14)**

2. 평소, 주말(또는 일하지 않는 날, 일하지 않는 전날)에 잠자리에 든 시각과 일어난 시각은 언제입니까?

① 잠자리에 든 시각 □□시 **(BP6_21)** □□분 **(BP6_22)**
② 일어난 시각 □□시 **(BP6_23)** □□분 **(BP6_24)**

1.2 Supplementary Figure S2 The Sleep Questionnaire (English Translated Version)

2-13. Mental Health

Item No.	Variable Type	Variable Name	Variable Description	Content
1	N(2)	BP16_11	(Aged 12 years or older) Time of going to bed on weekdays_hour	□□hour<br>88. Not applicable (pediatric)<br>99. Unknown, No response
1	N(2)	BP16_12	(Aged 12 years or older) Time of going to bed on weekdays_minute	□□minute<br>88. Not applicable (pediatric)<br>99. Unknown, No response
1	N(2)	BP16_13	(Aged 12 years or older) Time of waking up on weekdays_hour	□□hour<br>88. Not applicable (pediatric)<br>99. Unknown, No response
1	N(2)	BP16_14	(Aged 12 years or older) Time of waking up on weekdays_minute	□□minute<br>88. Not applicable (pediatric)<br>99. Unknown, No response
2	N(2)	BP16_21	(Aged 12 years or older) Time of going to bed on weekends_hour	□□hour<br>88. Not applicable (pediatric)<br>99. Unknown, No response
2	N(2)	BP16_22	(Aged 12 years or older) Time of going to bed on weekends_minute	□□minute<br>88. Not applicable (pediatric)<br>99. Unknown, No response
2	N(2)	BP16_23	(Aged 12 years or older) Time of waking up on weekends_hour	□□hour<br>88. Not applicable (pediatric)<br>99. Unknown, No response
2	N(2)	BP16_24	(Aged 12 years or older) Time of waking up on weekends_minute	□□minute<br>88. Not applicable (pediatric)<br>99. Unknown, No response
	N(4)*	Total_slp_wk	(Aged 12 years or older)	□□□□minutes<br>8888. Not applicable (pediatric)<br>9999. Unknown, No response
	N(4)*	Total_slp_we	(Aged 12 years or older)	□□□□minutes<br>8888. Not applicable (pediatric)<br>9999. Unknown, No response

*Derived variable

**Mental health well-being HNYR_ALL (YR represents the last two digits of the year) included in the DB.

Questionnaire

1. What time do you usually go to bed and wake up on weekdays (or workdays)?

① Bedtime	□□hour (BP6_11)	□□minute (BP6_12)
② Wake-up time	□□hour (BP6_13)	□□minute (BP6_14)

2. On a usual weekend (or on days off, the day before a day off), what time do you usually go to bed and wake up?

① Bedtime	□□hour (BP6_21)	□□minute (BP6_22)
② Wake-up time	□□hour (BP6_23)	□□minute (BP6_24)

1.3 Supplementary Table S1. Characteristics of the four adolescent sleep duration groups on weekdays and weekends*

	SWD/SWK† N=345 (29.6%) mean (SE)	LWD/SWK† N=255 (17.6%) mean (SE)	SWD/LWK† N=206 (17.7%) mean (SE)	LWD/LWK† N=500 (35.1%) mean (SE)	P value	LWD/SWK Vs. SWD/LWK P value
Age	**16.3 (0.08)**	**14.7 (0.13)**	**16.1 (0.11)**	**14.6 (0.11)**	**<0.001**	**<0.001**
Sex, Boys, weighted %	**51.7%**	**63.6%**	**39.5%**	**54.1%**	**<0.001**	**<0.001**
Overweight/obesity‡, weighted %	**26.7%**	**22.6%**	**14.8%**	**19.3%**	**0.017**	**0.012**
Sleep duration, hours						
TSD WD	**5.7 (0.04)**	**7.6 (0.04)**	**5.8 (0.04)**	**7.9 (0.04)**	**<0.001**	**<0.001**
TSD WK	**7.2 (0.05)**	**7.7 (0.06)**	**9.9 (0.08)**	**10.0 (0.07)**	**<0.001**	**<0.001**
TSD whole week	**6.1 (0.03)**	**7.6 (0.04)**	**7.0 (0.04)**	**8.5 (0.04)**	**<0.001**	**<0.001**
WK-E	**1.4 (0.06)**	**0.1 (0.07)**	**4.1 (0.09)**	**2.1 (0.07)**	**<0.001**	**<0.001**
Sleep time, hour:minutes						
BT WD	**01:07 (3.6 min)**	**23:37 (3.6 min)**	**01:02 (3.9 min)**	**23:21 (2.8 min)**	**<0.001**	**<0.001**
BT WK	**01:40 (4.8 min)**	**00:34 (4.8 min)**	**01:33 (5.4 min)**	**23:57 (3.8 min)**	**<0.001**	**<0.001**
WT WD	**06:51 (2.6 min)**	**07:14 (3.1 min)**	**06:52 (2.9 min)**	**07:16 (2.4 min)**	**<0.001**	**<0.001**
WT WK	**08:49 (5.5 min)**	**08:14 (5.2 min)**	**11:28 (6.5 min)**	**09:59 (5.7 min)**	**<0.001**	**<0.001**
Sedantary time (min)	**12.3 (0.2)**	**10.8 (0.2)**	**11.8 (0.2)**	**10.6 (0.2)**	**<0.001**	**0.002**
Total intake (kcal)	**2262.8 (65.7)**	**2200.3 (56.9)**	**2040.9 (76.2)**	**2113.7 (39.1)**	**0.015**	0.071
Physical activity						

Not at all	59.8%	58.1%	63.8%	65.0%	0.379	0.487
1 day	11.1%	16.1%	13.2%	9.0%		
2 days	9.5%	7.2%	7.8%	9.1%		
3 days	9.2%	4.2%	5.7%	7.0%		
4 days	2.8%	4.6%	0.8%	2.2%		
more than 5 days	7.6%	9.9%	8.7%	7.7%		
Household income						
Q1	8.3%	6.3%	12.2%	9.9%	0.055	0.257
Q2	14.7%	15.2%	15.7%	19.2%		
Q3	20.4%	26.5%	22.9%	22.9%		
Q4	25.5%	28.2%	30.6%	27.2%		
Q5	31.1%	23.8%	18.6%	20.7%		

Abbreviations: SWD, short sleep on weekdays; SWK, short sleep on weekends; LWD, long sleep on weekdays; LWK, long sleep on weekends; SE, standard error; TSD, total sleep duration; WD, weekdays; WK, weekends; BT, bedtime; WT, wake time; WK-E, weekend sleep extension. *, All data are weighted values considering the complex sample design. †, Categorization using the median values for TSD on weekdays and weekends. ‡, Categorization according to BMI z-score. Data are presented as mean (SE) or weighted %. Significant results are shown in bold. TSD whole week is calculated as (WD × 5 + WK × 2)/7
